# Multivariate control based on recurrent wavelet neural network for wastewater treatment process

**DOI:** 10.1371/journal.pone.0348671

**Published:** 2026-05-15

**Authors:** Yu Fang, Yin Su, You Li

**Affiliations:** College of Mechanical Engineering, Jiaxing University, Jiaxing, P. R. China; UniCamillus: Saint Camillus International University of Health and Medical Sciences, ITALY

## Abstract

The wastewater treatment process (WWTP), including multiple biochemical reactions, is a coupled and dynamic process. Thus, it is a challenge to achieve precise control of the WWTP. In order to address this issue, the self-organizing recurrent wavelet neural network controllers combined with the input mechanism (ISRWNNs) are proposed. First, the joint input mechanism is established for the coupling of dissolved oxygen (DO) and nitrate nitrogen (NO). Unlike the common multi-controller, the input of the proposed method takes into account both DO and NO errors to solve the coupling problem. Then, the self-organization algorithm of controller is proposed to automatically adjust the structure of the controllers for the dynamicity of WWTP. Furthermore, the stability of ISRWNN is analyzed through the Lyapunov stability theorem. Finally, the experimental results show that the proposed ISRWNN can obtain good control results of WWTP.

## 1. Introduction

Water pollution has become a major water environmental problem in the past decade. The wastewater treatment process (WWTP), which can improve water quality and save water resources [1], has become an important means to solve the water pollution problems. The main features of WWTP are as follows:1) The mechanism is complex, and it is difficult to establish the accurate kinetic model [2]. And the influent flow and components are easily affected by geographic and weather conditions, showing strong nonlinearity and dynamic characteristics. 2) The WWTP has multiple biochemical reaction processes, which are interrelated [3], leading to strong coupling problems between different control loops. When the control variables of one control loop change, it is easy to cause the other control variable to change. Then the smoothness of the control system is limited. Therefore, achieving stable and accurate control of the WWTP is still a difficult problem.

Due to the complex mechanism of WWTP, it is difficult to achieve the desired control performance by traditional control methods. To address this problem, some intelligent control methods have been proposed [4–8]. For example, the boundary-based predictive controller was proposed to keep the dissolved oxygen (DO) concentration in the activated sludge unit within the desired range [9]. The simulation results demonstrate that compared with the conventional on-off controller, the proposed controller can obtain superior control performance and stability when the system is disturbed. Chistiakova *et al*. designed a combined feedback and feedforward aeration controller to control DO to solve the time delay of WWTP [10]. In [11], the radial basis function (RBF) neural network controller was used to control the DO concentration to deal with the uncertainty of WWTP. Based on the RBF neural network, Han *et al*. developed the model predictive control (MPC) for DO concentration [12]. Compared to traditional feed-forward and PID control methods, the results show that intelligent methods have better control performance of DO. However, several unit processes and control parameters are contained in WWTP, which is a typical multivariable control system [13,14]. Since the WWTP is a typical multivariate process, these univariate control methods, which only consider DO, cannot guarantee the stable operation of the WWTP.

To solve the problem of univariate controller, some multivariate control methods have been designed for WWTP [15,16]. For instance, the MPC control combining two self-organizing RBF neural networks was proposed to control DO and nitrate nitrogen (NO) in the WWTP [17]. Based on MPC, Vege *et al*. proposed the combination of real-time optimization and MPC to control WWTP to improve effluent quality [18]. In [19], to control the activated sludge process, the recurrent neural network controllers were employed. The experimental results show that the proposed method can ensure the precision of control while reducing the energy cost, as well as reducing the nitrate concentration. Moreover, some other methods have been used for multivariate control in WWTP [20,21]. Among these methods, multi-neural network (NN) control can achieve accurate control of WWTP due to its powerful self-learning capability [22–24]. However, the structure of the controllers is fixed and difficult to adapt to different operating conditions.

To deal with the problem of fixed structure of NN controllers, which will affect the control performance, the self-constructing NN controller was designed for nonlinear system [25]. The controller incorporates the self-organization mechanism based on the mutual information to improve the control performance, which can automatically adjust the structure of controller. Wang *et al*. [26] used the self-organizing fuzzy NN control for uncertainty and unknown disturbances to improve the control accuracy of surface vehicles. The results prove that the adjustment mechanism of controller structure can adapt to the dynamic changes of the system and obtain superior control effects. However, the multi-NN controllers do not consider the coupling problem of the system [27,28]. Each control loop is independent, but the variables can affect each other, thus affecting the control performance of the system.

Based on the above analysis and discussion, the objective is to enhance the control performance of WWTP with respect to its dynamic characteristics and coupling problem. The recurrent wavelet neural network (RWNN), as a recurrent neural network which also combines the ability of wavelet analysis, can effectively improve the control accuracy of the network. Therefore, the RWNN is chosen as the controller in this paper. In the practical setting, the controllers with fixed structure are difficult to adapt to dynamically changing wastewater treatment process. Furthermore, the coupling between variables in WWTP will affect the performance of the controller. To solve these problems, the self-organizing RWNNs combined with an input mechanism (ISRWNNs) are designed to control the WWTP. The major benefits are as follows,

1. The input mechanism is designed to solve the coupling problem of WWTP. Unlike the input which only considers the error of a single variable, the input of ISRWNN considers the error of multiple variables and the variation of error.
2. Unlike controller with a fixed structure, the online self-organization algorithm is proposed based on the firing strength of wavelet neurons to deal with the dynamicity of WWTP. Then, the structure determination of ISRWNNs can be realized by automatically adjusting the number of wavelet nodes.
3. The stability of ISRWNNs when the structure is fixed and changed is guaranteed by the Lyapunov stability theorem respectively to ensure the practical application of controller.
4. Based on the benchmark simulation model no. 1 (BSM1) simulation platform, the control performance of ISRWNs is verified by comparing with other control methods. Experimental results show that the proposed ISRWNNs controller can effectively solve the problem of multivariate coupling in WWTP, and at the same time, the control accuracy can be improved.

The rest of this paper is organized as follows. Section 2 gives the details of the WWTP. In Section 3, the control method is presented. Experiment results are displayed in Section 4. Finally, Section 5 gives some conclusions.

## 2. WWTP

**Fig 1:**
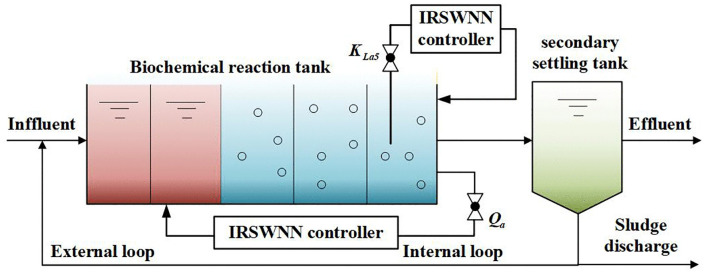
The model of WWTP.

The municipal wastewater treatment process is shown in Fig 1, which is mainly divided into two parts: the biochemical reaction tank and the secondary settling tank. In the secondary sedimentation tank, part of the biological denitrification of the wastewater is precipitated. In the biochemical reaction tank, a variety of biochemical reactions are carried out, with nitrification and denitrification reactions as the core processes. Among them, the concentration of DO plays an important role in the nitrification reaction; the NO has a significant impact on the rate of the denitrification reaction. In addition, too high or too low concentration of DO in the aerobic tank can inhibit the denitrification reaction and thus affect NO concentration. Low NO concentration can lead to an increase in aerobic tank aeration. It is indicated that there is a coupling between DO and NO. Therefore, how to achieve multivariate control of DO and NO and design controllers with decoupling performance are important to improve the efficiency of WWTP.

According to WWTP, the kinetic model of DO and NO is


$$\begin{array}{c} \left\{ \begin{array}{c} @l@\frac{dS_{NO,k}}{dt}=\frac{\text{1}}{V_{k}}\left(\left(Q_{a}+Q_{r}+Q_{\text{0}}\right)\left(Z_{k-1}-Z_{k}\right)+r_{k}V_{k}\right)\\ \frac{dS_{O,k}}{dt}=\frac{\text{1}}{V_{k}}\left(Q_{k-1}S_{O,k-1}+r_{k}V_{k}+K_{Lak}V_{k}\left(S_{O,sat}-S_{O,k}\right)-Q_{k}S_{O,k}\right) \end{array}\right. \end{array}$$ (1)

where $Q_{a}$ is the internal return flow of the second tank, $Q_{r}$ and $Q_{\text{0}}$ are the sludge return flow and inlet flow, $Z_{k}$, $V_{k}$, $r_{k}$ are the component concentration, volume and flow of the *k*th tank respectively, $Q_{k}$, $S_{o,k}$ and $K_{Lak}$ are the flow, DO concentration and oxygen conversion coefficient of the *k*th reaction tank respectively, $S_{o,sat}$ is the saturated concentration of DO. According to (1) and the mechanisms of nitrification and denitrifications reactions, the NO concentration depends on $Q_{a}$ and the DO concentration depends on $K_{Lak}$. Therefore, the $K_{La5}$ and $Q_{a}$ are chosen as the operating variables for the DO of fifth tank and NO of second tank.

## 3. The SRWNN controller combined with the input mechanism

**Fig 2:**
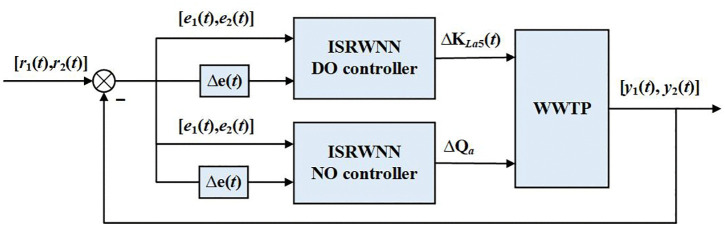
ISRWNNs control for WWTP.

The control methods is proposed in this section for WWTP, as shown in Fig 2. In the conventional multi-controllers, the coupling between variables is not considered and therefore the control accuracy is limited. To solve this problem, a control strategy combined with input mechanisms is introduced firstly, which improves the input of the controller by considering the error of multiple variables. Furthermore, the self-organizing algorithm is proposed to automatically adjust the structure of the controllers to adapt to different conditions of WWTP. The parameter learning algorithm is also presented. Finally, the stability proof of ISRWNN controller is given.

### 3.1 Control strategy

**Fig 3:**
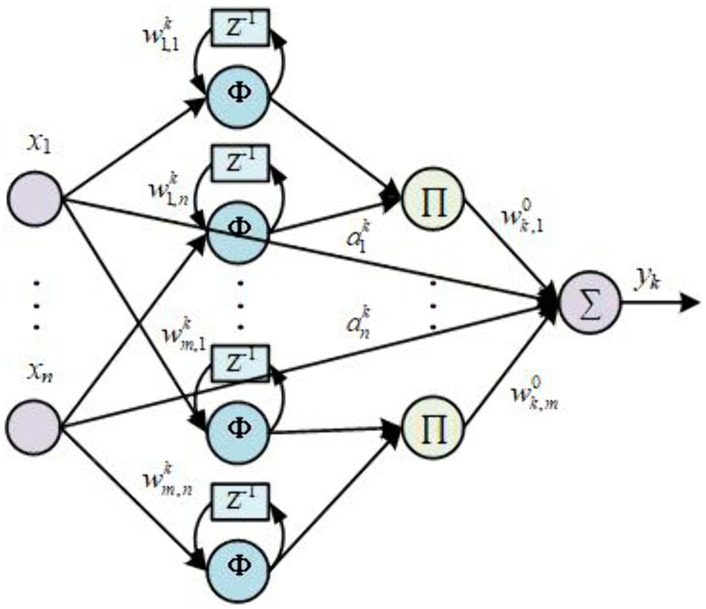
The structure of *k*th ISRWNN.


The structure of the ISRWNN controllers is shown in Fig 3. Suppose that there are *n* input neurons, *m*_*k*_ wavelet nodes and 1 output in the *k*th ISRWNN controller. Then the input vector of each ISRWNN controller is

$$\begin{array}{c} 𝐱\left(t\right)=\left[e_{DO}\left(t\right),\Delta e_{DO}\left(t\right),e_{NO}\left(t\right),\Delta e_{NO}\left(t\right)\right] \end{array}$$ (2)

where $e_{DO}\left(t\right)=S_{DO,set}-S_{DO}$ and $e_{NO}\left(t\right)=S_{NO,set}-S_{NO}$ are the errors of the DO and NO, $S_{DO,set}$ and $S_{NO,set}$ are the set-point of DO and NO, $S_{DO}$ and $S_{NO}$ are the value of DO and NO concentration at time *t*, respectively.$\Delta e_{DO}\left(t\right)$ and $\Delta e_{NO}\left(t\right)$ are the variation of error. The errors of DO and NO are incorporated into the inputs of both controllers. The NO concentration error is used to preemptively adjust the DO controller output to counteract the coupling effect from the NO loop to the DO loop. Conversely, the DO error similarly compensates the NO control action. This bidirectional compensation is realized through a decoupling network integrated into the ISRWNN framework, which dynamically adjusts control signals based on real-time error variations. From a control theory viewpoint, the decoupling is achieved through coupling error compensation embedded in the multi-variable input structure. Specifically, each controller is designed not only to track its own reference but also to actively compensate for the coupling effects originating from other input variables. This is realized by constructing a set of compensating signals that cancel the interaction terms in the system dynamics [29–31].

Then the operating variables $K_{La5}\left(t\right)$ and $Q_{a}\left(t\right)$ can be calculated as


$$\begin{array}{c} \left\{ \begin{array}{c} @l@K_{La5}\left(t\right)=K_{La5}(t-1)+\Delta K_{La5}\left(t\right)\\ Q_{a}\left(t\right)=Q_{a}(t-1)+\Delta Q_{a}\left(t\right) \end{array}\right. \end{array}$$ (3)

where $\Delta K_{La5}\left(t\right)=y_{\text{1}}\left(t\right)$ and $\Delta Q_{a}\left(t\right)=y_{\text{2}}\left(t\right)$ are the outputs of the two ISRWNN controllers, respectively. Furthermore, the $\Delta K_{La5}\left(t\right)$ and $\Delta Q_{a}\left(t\right)$ are the variation of $K_{La5}\left(t\right)$ and $Q_{a}\left(t\right)$. For the *k*th controller, the expression is as follows.


$$\begin{array}{c} y_{k}\left(t\right)=\sum_{j=1}^{m_{k}}\overset{\text{0}}{\underset{k,j}{w}}\left(t\right)\overset{\left(3\right)}{\underset{k,j}{u}}\left(t\right)+\sum_{i=1}^{n}\overset{k}{\underset{i}{a}}\left(t\right)x_{i}\left(t\right)\;(k=1,2) \end{array}$$ (4)

where $\overset{\text{0}}{\underset{k,j}{w}}\left(t\right)$ is the output weight of the *j*th wavelet node, $\overset{k}{\underset{i}{a}}\left(t\right)$ is the input weight of *i*th input neuron, $x_{i}\left(t\right)$ is the *i*th input at time *t*, $\overset{\left(3\right)}{\underset{k,j}{u}}\left(t\right)$ is the output of *j*th wavelet node which can be calculated as


$$\begin{array}{c} \overset{\left(3\right)}{\underset{k,j}{u}}\left(t\right)=\prod_{i=1}^{n}\overset{k}{\underset{i,j}{u}}\left(t\right)=\prod_{i=1}^{n}\left[-\overset{k}{\underset{i,j}{\theta }}\left(t\right)exp\left(-\frac{\text{1}}{\text{2}}{\left(\overset{k}{\underset{i,j}{\theta }}\left(t\right)\right)}^{\text{2}}\right)\right] \end{array}$$ (5)

$$\begin{array}{c} \overset{k}{\underset{i,j}{\theta }}\left(t\right)=\frac{\overset{k}{\underset{i,j}{h}}\left(t\right)-\overset{k}{\underset{i,j}{b}}\left(t\right)}{\overset{k}{\underset{i,j}{c}}\left(t\right)} \end{array}$$ (6)

$$\begin{array}{c} \overset{k}{\underset{i,j}{h}}\left(t\right)=x_{i}\left(t\right)+\overset{k}{\underset{j,i}{w}}\left(t\right)\overset{k}{\underset{i,j}{u}}(t-1) \end{array}$$ (7)

where $\overset{k}{\underset{i,j}{u}}\left(t\right)$ is the output of the mother wavelet neuron at time *t*, $\overset{k}{\underset{i,j}{b}}\left(t\right)$ and $\overset{k}{\underset{i,j}{c}}\left(t\right)$ are the translation and dilation coefficient respectively, $\overset{k}{\underset{j,i}{w}}\left(t\right)$ is the weight of the feedback loop, $\overset{k}{\underset{i,j}{u}}(t-1)$ is the output of the mother wavelet neuron at time (*t*-1),.

*Remark 1*. Different with the conventional multi-controller methods, the input of the proposed ISRWNN controller considers both DO and NO errors. Compared with considering only the error of the single variable, it can effectively improve the decoupling ability of the controller.

### 3.2. Structure adjustment mechanism

In neural network control, the selection of appropriate network structure is an important issue, which will affect the control performance. Therefore, the growing and pruning mechanism of wavelet base is proposed to automatically determine the structure of the controller for different operating conditions. The firing strength of the wavelet neuron serves as the precise quantitative measure of the alignment between input signals and the localized features encoded by wavelet nodes [30]. Compared to other abstract internal signals, the firing strength offers superior interpretability. Moreover, due to the localized response characteristics of wavelet functions, only a small subset of nodes is significantly activated when processing specific inputs. This inherent sparsity enables rapid and accurate identification of both critical and redundant nodes, thereby substantially improving the computational efficiency of structural self-adaptation. Consequently, leveraging firing strength to guide the structural evolution of ISRWNN not only enhances the transparency and trustworthiness of the network but also ensures its simplicity and efficiency during dynamic learning. The expression of firing strength is shown as follows.


$$\begin{array}{c} D_{k,j}=\left|\overset{\left(3\right)}{\underset{k,j}{u}}\right| \end{array}$$ (8)

where $\overset{\left(3\right)}{\underset{k,j}{u}}\left(t\right)$ is the output of *j*th wavelet node of *k*th controller. Then, the structure adjustment algorithm is introduced as follows.

*1) Growing Phase*: In the growing phase, the neural network generates new nodes to make full use of the effective wavelet base. The new wavelet neuron is added when the ratio of the firing strength meets the following requirements


$$\begin{array}{c} P_{k,j}=\frac{D_{k,j}}{\overset{m_{k}}{\underset{j=1}{\sum }}D_{k,j}}>P_{k,max} \end{array}$$ (9)

where $P_{k,max}$ is the threshold of *k*th ISRWNN during the growing phase. The initial parameters of newly added wavelet node are set as


$$\begin{array}{c} \left\{ \begin{array}{c} @l@\overset{\text{0}}{\underset{k,m+1}{w}}\left(t\right)=\left(d_{k}\left(t\right)-y_{k}\left(t\right)\right){\left(\overset{\left(3\right)}{\underset{k,m+1}{u}}\left(t\right)\right)}^{-1}\\ \overset{k}{\underset{i,m+1}{b}}\left(t\right)=r_{\text{1}},\overset{k}{\underset{i,m+1}{c}}\left(t\right)=r_{\text{2}},\overset{k}{\underset{i,m+1}{w}}\left(t\right)=r_{\text{3}} \end{array}\right. \end{array}$$ (10)

where $d_{k}\left(t\right)$ is the desired output, $\overset{\left(3\right)}{\underset{k,m+1}{u}}\left(t\right)$ is the output of (*m* + 1)th neuron, $\left\{r_{\text{1}},r_{\text{2}},r_{\text{3}}\right\}$ are randomly selected from the range of [−1, 1].

*2) Pruning Phase*: In the pruning phase, the redundant nodes will be removed. The *l*th wavelet node will be deleted if the following condition is satisfied


$$\begin{array}{c} P_{k,j}=\frac{D_{k,j}}{\overset{m_{k}}{\underset{j=1}{\sum }}D_{k,j}}<P_{k,min} \end{array}$$ (11)

where $P_{k,min}$ is the pruning threshold of the *k*th controller. If the *l*th wavelet neuron is deleted, the output weight of the l′th wavelet node with the shortest Euclidean distance from the *l*th neuron will be adjusted as follow.


$$\begin{array}{c} \overset{\text{0}}{\underset{k,l\prime }{w}}\prime \left(t\right)=\overset{\text{0}}{\underset{k,l\prime }{w}}\left(t\right)+\overset{\text{0}}{\underset{k,l}{w}}\left(t\right)\overset{\left(3\right)}{\underset{k,l}{u}}\left(t\right){\left(\overset{\left(3\right)}{\underset{k,l\prime }{u}}\left(t\right)\right)}^{-1} \end{array}$$ (12)

where $\overset{\text{0}}{\underset{k,l\prime }{w}}\left(t\right)$ and $\overset{\left(3\right)}{\underset{k,l\prime }{u}}\left(t\right)$ are the output weight and output of the l′th wavelet node before deleting, $\overset{\text{0}}{\underset{k,l}{w}}\left(t\right)$ and $\overset{\left(3\right)}{\underset{k,l}{u}}\left(t\right)$ are the output weight and output of the *l*th wavelet node before pruning, $\overset{\text{0}}{\underset{k,l\prime }{w}}\prime \left(t\right)$ is the output weight of the l′th wavelet node after deleting.

*Remark* 2. Two ISRWNN controllers are independent of each other, and the structure adjustment is carried out simultaneously. Through the self-organizing algorithm, the controllers can automatically adjust their structures for different input conditions to further improve the control performance.

### 3.3. Parameter learning mechanism

The controller based on an offline parameter training algorithm is difficult to adapt to real-time changes in WWTP. Thus, the parameters of ISRWNN are updated online based on the gradient descent method. The cost function of the controller is set as


$$\begin{array}{c} E_{k}\left(t\right)=\frac{\text{1}}{\text{2}}\overset{\text{2}}{\underset{k}{e}}\left(t\right)=\frac{\text{1}}{\text{2}}{\left(d_{k}\left(t\right)-y_{k}(t)\right)}^{\text{2}} \end{array}$$ (13)

where $e_{k}\left(t\right)$ is the error of *k*th controller. For brevity, Ω(t) is used to denote the ensemble of parameters, i.e., $\Omega_{k}\left(t\right)=\left[\overset{k}{\underset{j,i}{w}},\overset{k}{\underset{j,i}{b}},\overset{k}{\underset{j,i}{c}},\overset{\text{0}}{\underset{k,j}{w}},\overset{k}{\underset{i}{a}}\right]$. Then the parameters can be learned as follows.


$$\begin{array}{c} \Omega_{k}(t+1)=\Omega_{k}\left(t\right)-\lambda_{k}\frac{\partial E_{k}\left(t\right)}{\partial \Omega_{k}\left(t\right)} \end{array}$$ (14)

where $\lambda_{k}=\left[\lambda_{1,k},\lambda_{2,k},\lambda_{3,k},\lambda_{4,k},\lambda_{5,k}\right]$ are the adaptive learning rates, which are expressed as follows.


$$\begin{array}{c} \lambda_{k}=\alpha {\left[{\left(\frac{\partial e_{k}\left(t\right)}{\partial \Omega_{k}\left(t\right)}\right)}^{T}\left(\frac{\partial e_{k}\left(t\right)}{\partial \Omega_{k}\left(t\right)}\right)+\eta \right]}^{-1} \end{array}$$ (15)

where α is the coefficient with value in the range (0,2/5), η is a positive number close to 0.

### 3.3 Control process of WWTP

For a clearer representation, the specific control process of WWTP is shown in Table 1. During the control process, the parameters are continuously learned to achieve the control accuracy requirements. On the other hand, according to the structure adjustment algorithm, the wavelet bases can be added and removed to obtain the appropriate ISRWNN structure.

**Table 1:** The control process of WWTP.

Initialization: Initialize two ISRWNN controllers with hidden nodes as 5, the parameters of controllers $\Omega_{k}\left(t\right)$. Set the parameters $P_{k,max}$, $P_{k,min}$, α and η.
for $t=1:t_{max}$ Calculate the outputs of ISRWNN $\Delta K_{La5}\left(t\right)$ and $\Delta Q_{a}\left(t\right)$ Update $\Omega_{\text{1}}\left(t\right)$ and $\Omega_{\text{2}}\left(t\right)$ Calculate the $D_{k,j}$ and $P_{k,j}$ of wavelet neuron
If the $P_{k,j}$ satisfies (9) Add one new wavelet neuron with initial parameters as (10) If the $P_{k,j}$ satisfies (11) Delete the neuron and adjust the parameters of its nearest wavelet neuron as shown in (12) End End

### 3.4 Stability analysis

In the design of the control system, the stability of the controller is very important for practical applications. Therefore, the stability of the ISRWNN controller will be verified in terms of both fixed and changed structures. Although the wastewater treatment process itself is continuous in nature, the control signals are sampled and updated at discrete time instants due to the digital implementation of the ISRWNN controller. Therefore, the closed-loop system should be treated as a sampled-data system, and its stability analysis is conducted within a discrete-time framework.

*Theorem 1.* Assume that the number of wavelet nodes of the ISRWNNs controller is $m_{\text{1}}$ and $m_{\text{2}}$ respectively, and the adaptive learning rates of the parameter learning algorithm are set to (15). If the regulation parameter α of $\lambda_{k}$ satisfies the following condition


$$\begin{array}{c} 0<\alpha <2/5 \end{array}$$ (16)

Then the stability of the fixed structure ISRWNN controllers can be guaranteed.

*Proof*. Consider the Lyapunov function of the control system at time *t* with fixed structure as


$$\begin{array}{c} V\left(t\right)=\frac{\text{1}}{\text{2}}\left(\overset{\text{2}}{\underset{\text{1}}{e}}\left(t)+\overset{\text{2}}{\underset{\text{2}}{e}}(t\right)\right) \end{array}$$ (17)

where $e_{\text{1}}\left(t\right)$ and $e_{\text{2}}\left(t\right)$ are the control errors of the ISRWNNs controller, respectively. Then the change in (17) is


$$\begin{array}{c} \Delta V\left(t\right)=V(t+1)-V\left(t\right)=\frac{\text{1}}{\text{2}}\sum_{k=1}^{\text{2}}\left(\overset{\text{2}}{\underset{k}{e}}\left(t+1)-\overset{\text{2}}{\underset{k}{e}}(t\right)\right) \end{array}$$ (18)

According to [32], the difference of control error can be expressed by


$$\begin{array}{c} \Delta e_{k}\left(t\right)=e_{k}(t+1)-e_{k}\left(t\right)\approx \left[\frac{\partial e_{k}\left(t\right)}{\partial \Omega_{k}\left(t\right)}\right]\Delta \Omega_{k}\left(t\right) \end{array}$$ (19)

Based on (14) and (15), since ${\left(\frac{\partial e_{k}\left(t\right)}{\partial \Omega_{k}\left(t\right)}\right)}^{T}\left(\frac{\partial e_{k}\left(t\right)}{\partial \Omega_{k}\left(t\right)}\right)>0$, and η is a positive number close to 0, we have


$$\begin{array}{c} \left[\frac{\partial e_{k}\left(t\right)}{\partial \Omega_{k}\left(t\right)}\right]\Delta \Omega_{k}\left(t\right)=-\lambda e_{k}\left(t\right){\left[\frac{\partial e_{k}\left(t\right)}{\partial \Omega_{k}\left(t\right)}\right]}^{T}\left[\frac{\partial e_{k}\left(t\right)}{\partial \Omega_{k}\left(t\right)}\right]=-5\alpha e_{k}\left(t\right) \end{array}$$ (20)

Then (20) can be substituted into (18), it has


$$\begin{array}{c} \begin{array}{c} \Delta V\left(t\right)=\frac{\text{1}}{\text{2}}\sum_{k=1}^{\text{2}}\left(2e_{k}\left(t\right)\Delta e_{k}\left(t\right)+{\left(\Delta e_{k}\left(t\right)\right)}^{\text{2}}\right)\\ \text{=}\frac{\text{1}}{\text{2}}\left(10\varepsilon -25\varepsilon^{\text{2}}\right)\left(\overset{\text{2}}{\underset{\text{1}}{e}}\left(t\right)+\overset{\text{2}}{\underset{\text{2}}{e}}\left(t\right)\right) \end{array} \end{array}$$ (21)

Based on (17), we can obtain that V(t)>0. Therefore, when 0<α<2/5, substituting into (21), it has 0<V(t+1)<V(t). Then the Theorem 1 can be proved.

*Theorem* 2. At time *t*, suppose that the structure of the kth ISRWNN changes, i.e., the number of wavelet nodes changes. If the expression of λ is as (15), the stability of ISRWNN can also be obtained.

*Proof*. At time *t*, when a new wavelet node is added to the *k*th ISRWNN, the number of wavelet nodes is $\left(m_{k}+1\right)$. Based on (10), the control error is calculated as follows,


$$\begin{array}{c} \begin{array}{c} \overset{\prime }{\underset{k}{e}}\left(t\right)=d_{k}\left(t\right)-\sum_{j=1}^{m_{k}+1}\overset{\text{0}}{\underset{k,j}{w}}\left(t\right)\overset{\left(3\right)}{\underset{k,j}{u}}\left(t\right)+\sum_{i=1}^{n}\overset{k}{\underset{i}{a}}\left(t\right)x_{i}\left(t\right)\\ \text{=}d_{k}\left(t\right)-\sum_{j=1}^{m_{k}}\overset{\text{0}}{\underset{k,j}{w}}\left(t\right)\overset{\left(3\right)}{\underset{k,j}{u}}\left(t\right)+\sum_{i=1}^{n}\overset{k}{\underset{i}{a}}\left(t\right)x_{i}\left(t\right)-\overset{\text{0}}{\underset{k,m_{k}+1}{w}}\left(t\right)\overset{\left(3\right)}{\underset{k,m_{k}+1}{u}}\left(t\right)\\ \text{=}d_{k}\left(t\right)-y_{k}\left(t\right)-\left(d_{k}\left(t\right)-y_{k}\left(t\right)\right){\left(\overset{\left(3\right)}{\underset{k,m_{k}+1}{u}}\left(t\right)\right)}^{-1}\overset{\left(3\right)}{\underset{k,m_{k}+1}{u}}\left(t\right)\text{=0} \end{array} \end{array}$$ (22)

Meanwhile, when the *l*th wavelet neuron is deleted, the number of hidden nodes after pruning is $\left(m_{k}-1\right)$. Based on (12), the approximate error of *k*th ISRWNN is calculated as


$$\begin{array}{c} \begin{array}{c} \overset{\prime }{\underset{k}{e}}\left(t\right)=d_{k}\left(t\right)-\sum_{j=1}^{m_{k}-1}\overset{\text{0}}{\underset{k,j}{w}}\left(t\right)\overset{\left(3\right)}{\underset{k,j}{u}}\left(t\right)-\sum_{i=1}^{n}\overset{k}{\underset{i}{a}}\left(t\right)x_{i}\left(t\right)\\ \text{=}d_{k}\left(t\right)-\sum_{j=1,j\neq l\prime }^{m_{k}-1}\overset{\text{0}}{\underset{k,j}{w}}\left(t\right)\overset{\left(3\right)}{\underset{k,j}{u}}\left(t\right)-\overset{\text{0}}{\underset{k,l\prime }{w}}\left(t\right)\overset{\left(3\right)}{\underset{k,l\prime }{u}}\left(t\right)-\sum_{i=1}^{n}\overset{k}{\underset{i}{a}}\left(t\right)x_{i}\left(t\right)\\ \text{=}d_{k}\left(t\right)-\sum_{j=1,j\neq l\prime }^{m_{k}-1}\overset{\text{0}}{\underset{k,j}{w}}\left(t\right)\overset{\left(3\right)}{\underset{k,j}{u}}\left(t\right)-\left(\overset{\text{0}}{\underset{k,l\prime }{w}}\left(t\right)+\overset{\text{0}}{\underset{k,l}{w}}\left(t\right)\overset{\left(3\right)}{\underset{k,l}{u}}\left(t\right){\left(\overset{\left(3\right)}{\underset{k,l\prime }{u}}\left(t\right)\right)}^{-1}\right)\overset{\left(3\right)}{\underset{k,l\prime }{u}}\left(t\right)-\sum_{i=1}^{n}\overset{k}{\underset{i}{a}}\left(t\right)x_{i}\left(t\right)\\ \text{=}d_{k}\left(t\right)-\sum_{j=1}^{m_{k}}\overset{\text{0}}{\underset{k,j}{w}}\left(t\right)\overset{\left(3\right)}{\underset{k,j}{u}}\left(t\right)-\sum_{i=1}^{n}\overset{k}{\underset{i}{a}}\left(t\right)x_{i}\left(t\right)\text{=}e_{k}\left(t\right) \end{array} \end{array}$$ (23)

According to Theorem 1, if the α is selected from the range of (0,2/5), Theorem 2 can be proved.

*Remark* 3. The stability proof relies on the ideal assumption that the error conforms to Equation (19). In practical operation, however, WWTP is prone to various disturbances, meaning actual errors are far more complex. Therefore, the stability of proposed method is only assured under disturbance-free or weak-disturbance conditions.

## 4. Simulation results and discussion

To validate the control performance of ISRWNNs on WWTP, the experiments are conducted on the BSM1 platform. In the experiments, the set-points of DO and NO concentrations are set as constant and step values, respectively. Furthermore, the parameters of the ISRWNN are determined by the grid search method, which is based on [33]. The grid search is performed over the following ranges: *P*_max_ from 0.4 to 0.8 with a step size of 0.1; *P*_min_ from 0.01 to 0.1 with a step size of 0.01; and α from 0 to 0.4 with a step size of 0.05. The optimal parameter set is selected to minimize the control error of DO concentration. Then the parameters of the two ISRWNNs are chosen as $P_{1,max}=0.4$, $P_{2,max}=0.5$, $P_{1,min}=0.01$, $P_{2,min}=0.02$, α=0.25 and η=0.001. To further verify the control performance, the ISRWNN is compared with the RWNN, RWNN combined with input mechanism (IRWNN), recurrent fuzzy neural network (RFNN), and RBF neural network (RBFNN) controller. Moreover, the integral of absolute error (IAE), integral of squared error (ISE), and maximal deviation from set point (${\text{Dev}}^{max}$) are chosen to evaluate the control performance of DO and NO concentration as follows.


$$\begin{array}{c} \text{IAE}=\frac{\text{1}}{N}\sum_{t=1}^{t_{max}}\left|d\left(t\right)-y\left(t\right)\right| \end{array}$$ (24)

$$\begin{array}{c} \text{ISE}=\frac{\text{1}}{N}\sum_{t=1}^{t_{max}}{\left(d\left(t\right)-y\left(t\right)\right)}^{\text{2}} \end{array}$$ (25)

$$\begin{array}{c} {\text{Dev}}^{max}=max\left\{\left|d\left(t\right)-y\left(t\right)\right|\right\} \end{array}$$ (26)

where *N* is the total number of samples. The smaller IAE, ISE and ${\text{Dev}}^{max}$ represent better control performance.

In the experiment, 14 days of data are selected for selection under both rainy and rainstorm conditions. Under rainy conditions, the first 7 days of data are dry weather data, and days 9–11 are data from a long period of continuous rainfall. Under rainstorm conditions, sudden downpours on days 10 and 12 are simulated with increased water intake. Based on the BSM1 simulation platform with a sampling period of 15 minutes, the data from the last 7 days are applied to calculate the control indicators.

### 4.1 Constant set-point of DO and NO concentration

**Fig 4:**
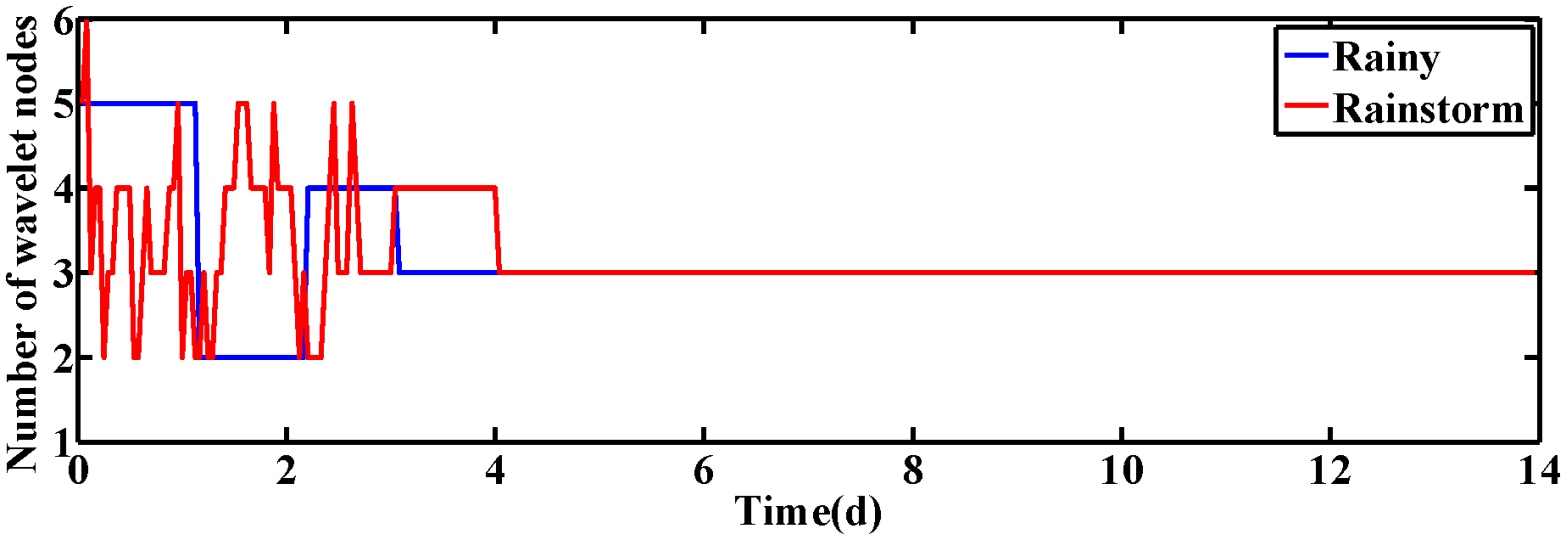
The number of wavelet nodes of DO controller.

**Fig 5:**
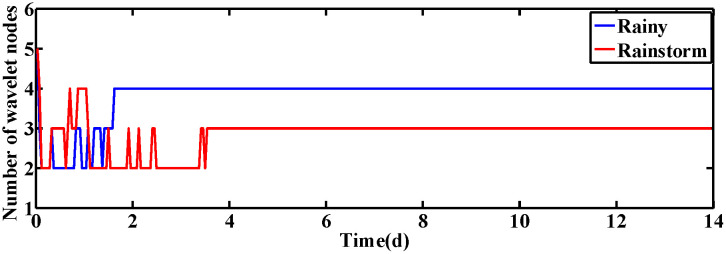
The number of wavelet nodes of NO controller.

In this subsection, the performance of ISRWNNs is measured at constant setting values, i.e., $S_{DO,set}=2mg\cdot L^{-1}$ and $S_{NO,set}=1mg\cdot L^{-1}$. The variation of wavelet neurons of DO and NO controllers are shown in Figs 4 and 5, respectively. Obviously, the wavelet nodes are able to adjust automatically and finally converge under different operating conditions, indicating the effectiveness of the self-organization algorithm. This phenomenon is consistent with the proof of Theorem 2.

**Fig 6:**
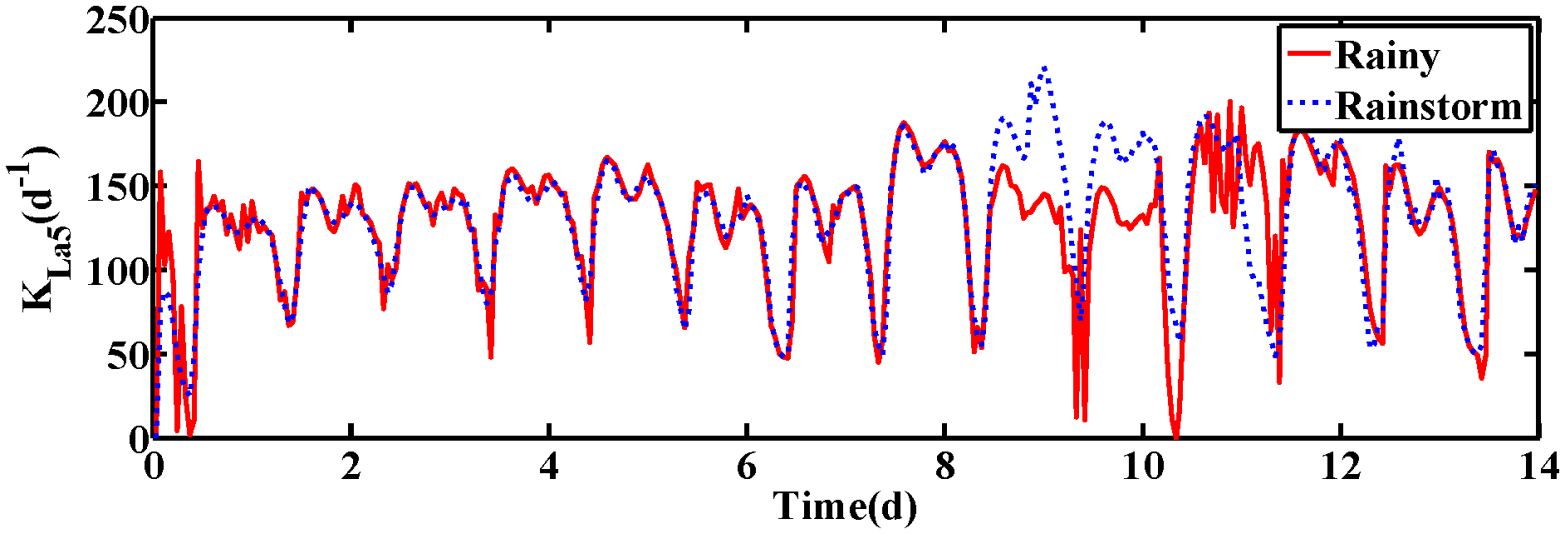
The value of $K_{La5}$ under two conditions.

**Fig 7:**
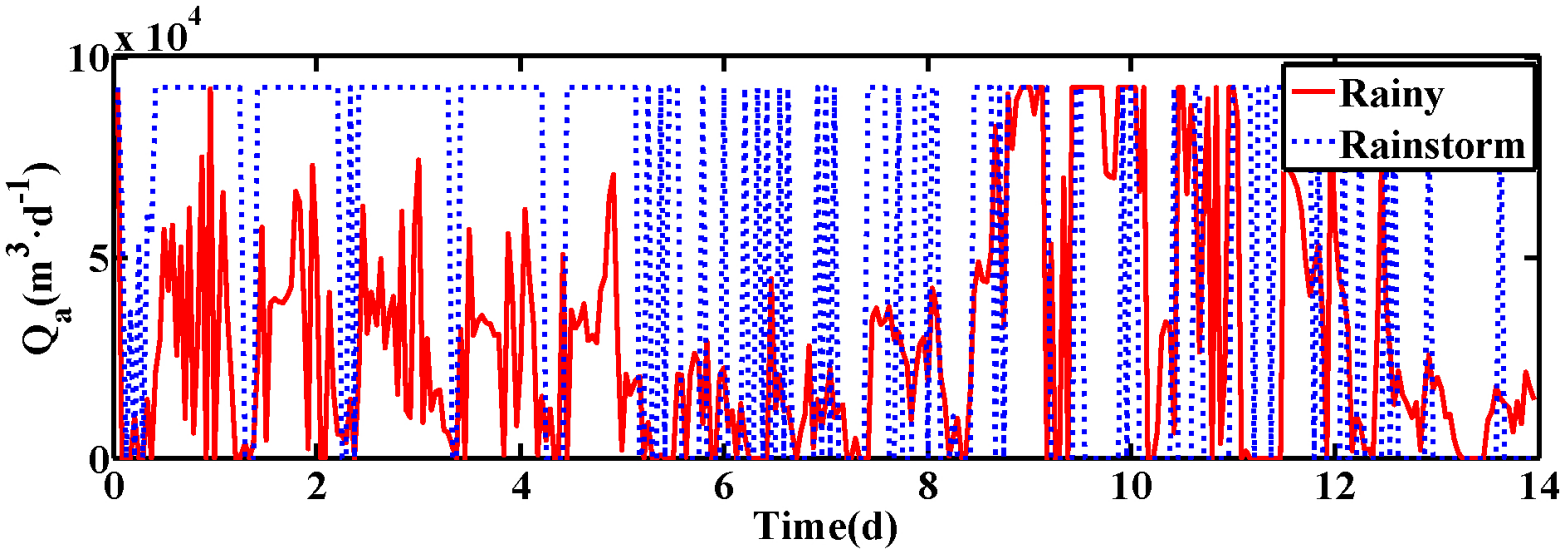
The value of $Q_{a}$ under two conditions. **(a)** Control results of DO and NO concentration. **(b)** Control errors of DO and NO concentration.

**Fig 8:**
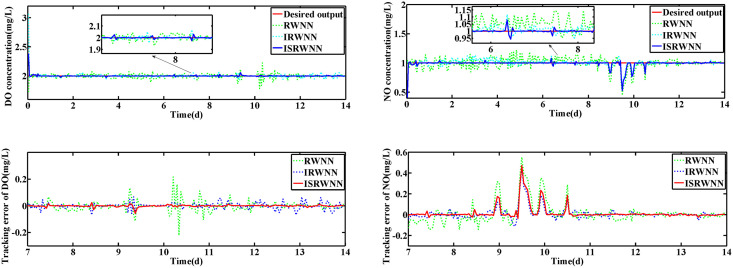
Control results under rainy condition during set-point fixed. **(a)** Control results of DO and NO concentration. **(b)** Control errors of DO and NO concentration.

**Fig 9:**
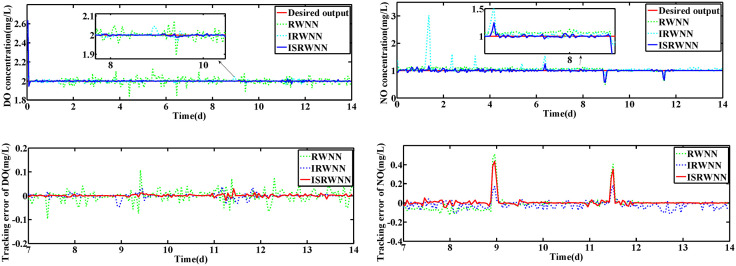
Control results under rainstorm condition during set-point fixed.

The changes of $K_{La5}$ and $Q_{a}$ are shown in Figs 6 and 7, which lead to the variation of DO and NO concentration. The control results under rainy and rainstorm conditions are displayed in Figs 8 and 9, respectively. To reflect the control results more clearly, it can be seen from the enlarged section that the control effect of the IRWNN is better compared to the basic RWNN controller, proving that the input adjustment mechanism can improve the accuracy of control. Otherwise, under the same simulation sampling period, the total computation time for the ISRWNN is 15 seconds, compared to 9 seconds for the RWNN. This result indicates that the additional overhead of the self-organizing mechanism is acceptable and does not incur a significant time penalty. Meanwhile, the ISRWNN has the smallest control error among the three control methods. It can be concluded that the structure of the controller will affect the control accuracy, which is avoided by the self-organization algorithm.

To better evaluate the control performance of ISRWNN, the comparison results of different controllers are summarized in Table 2, where *No*. indicates the number of wavelet/hidden neurons of the controller. It can be observed that the ISRWNN controllers have the smallest IAE, ISE and ${\text{Dev}}^{max}$ for both DO concentration and NO concentration. Furthermore, the control IAE of IRWNN is smaller than that of RWNN, and the difference in control error between DO and NO is smaller, which suggests that the input adjustment can solve the coupling problem of DO and NO concentration.

**Table 2:** Comparison of different control methods for fixed set-point.

Condition	Controller	DO				NO			
		No.	IAE	ISE	Dev^max^	No.	IAE	ISE	Dev^max^
Rainy	ISRWNN	3	0.0083	2.79 × 10^−4^	0.0683	4	0.0141	1.91 × 10^−3^	0.3522
	IRWNN	3	0.0097	8.53 × 10^−4^	0.1665	3	0.017	7.46 × 10^−3^	0.4473
	RWNN	3	0.0134	9.38 × 10^−4^	0.1841	3	0.0281	9.39 × 10^−3^	0.5089
	RFNN	6	0.0184	2.01 × 10^−3^	0.2749	6	0.3950	5.33 × 10^−2^	1.9522
	RBFNN	5	0.0198	2.43 × 10^−3^	0.3742	5	0.4613	6.36 × 10^−2^	1.9978
Rainstorm	ISRWNN	3	0.0029	5.34 × 10^−5^	0.0459	3	0.0093	1.85 × 10^−3^	0.3339
	IRWNN	3	0.0046	1.57 × 10^−4^	0.1167	3	0.0109	1.92 × 10^−3^	0.4281
	RWNN	3	0.0078	4.33 × 10^−4^	0.0929	3	0.0142	2.36 × 10^−3^	0.3848
	RFNN	6	0.0081	6.31 × 10^−4^	0.1637	6	0.3715	5.18 × 10^−2^	1.1077
	RBFNN	5	0.0096	7.41 × 10^−4^	0.2913	5	0.4303	3.96 × 10^−2^	1.1977

### 4.2 Changing set-point of DO and NO concentration

In order to further verify the decoupling control performance of ISRWNN (i.e., the control effect of the system on another variable when one variable is changed), the step-change DO concentration and NO concentration set-points are used and set as follows.


$$\begin{array}{c} S_{DO,set}=\left\{ \begin{array}{c} @l@1.8mg\cdot L^{-1}\;\;8\leq t<9\\ 2.2mg\cdot L^{-1}\;\;9\leq t<10\\ 2mg\cdot L^{-1}\;\;otherwise \end{array}\right. \end{array}$$ (27)

$$\begin{array}{c} S_{NO,set}=\left\{ \begin{array}{c} @l@0.9mg\cdot L^{-1}\;\;11\leq t<12\\ 1.1mg\cdot L^{-1}\;\;12\leq t<13\\ 1mg\cdot L^{-1}\;\;otherwise \end{array}\right. \end{array}$$ (28)

where t=[0,14].

**Fig 10:**
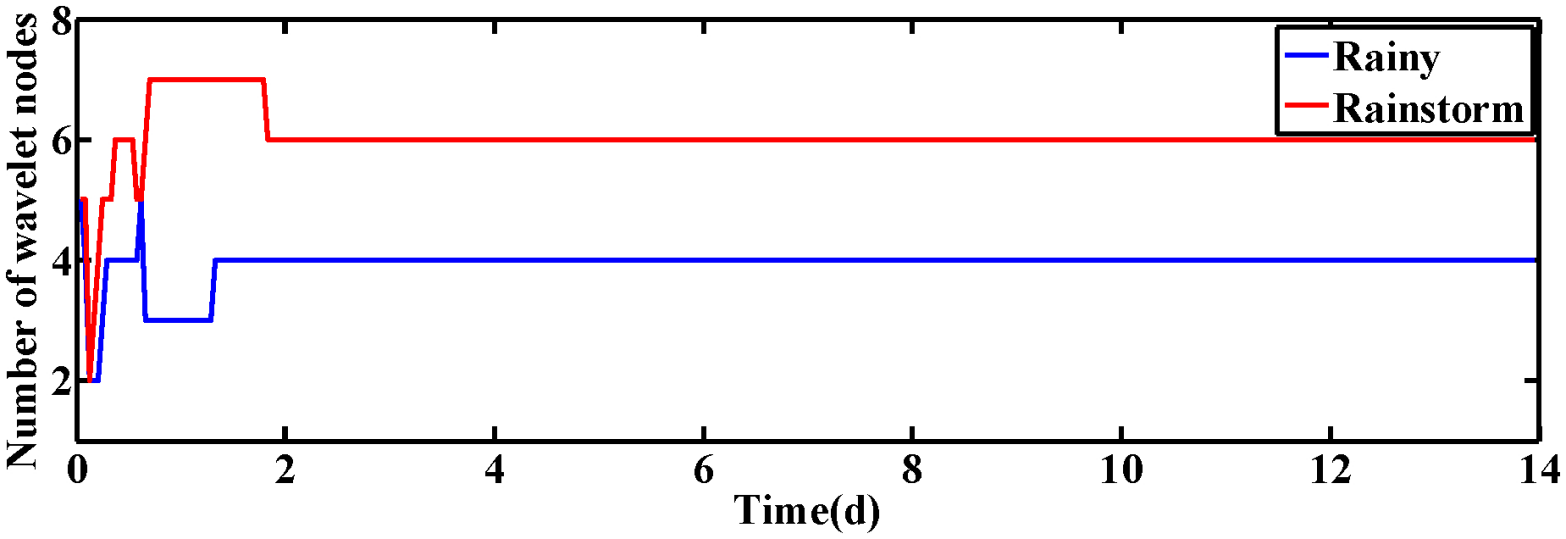
The number of wavelet nodes of DO controller.

**Fig 11:**
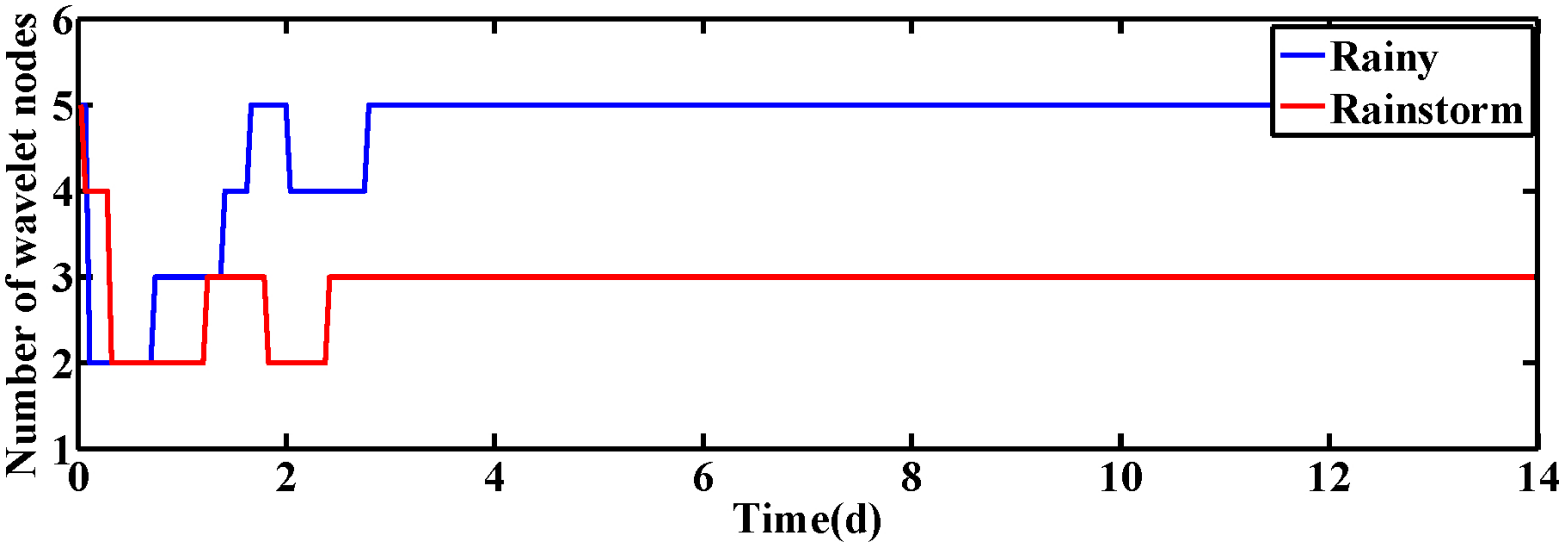
The number of wavelet nodes of NO controller.

When the desired values of DO and NO concentration are varied, the changes in the wavelet nodes of ISRWNNs under different conditions are shown in Figs 10 and 11, respectively. It can be seen that the number of wavelet nodes keeps changing as time advances, and finally converges to a fixed value and remains constant. Simulation results show that the self-organizing mechanism is also able to automatically adjust the structure of the controller according to different conditions when the set values change dynamically.

**Fig 12:**
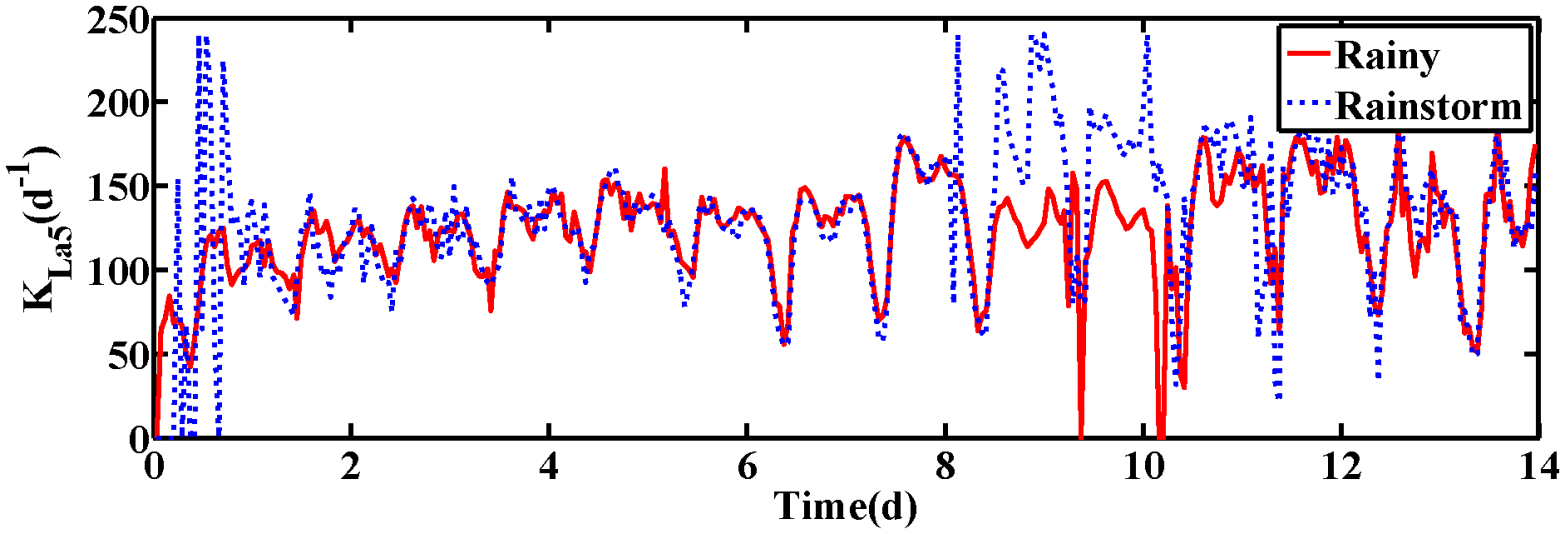
The value of $K_{La5}$ under two conditions.

**Fig 13:**
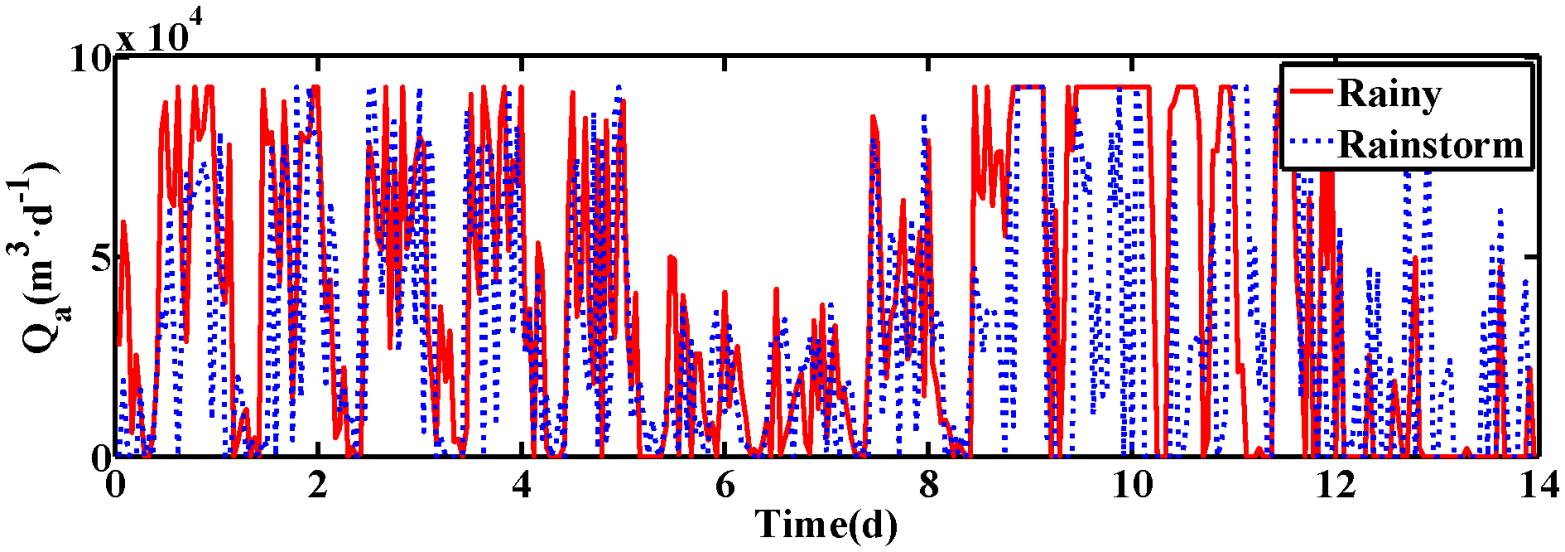
The value of $Q_{a}$ under two conditions. **(a)** Control results of DO and NO concentration. **(a)** Control errors of DO and NO concentration.

**Fig 14:**
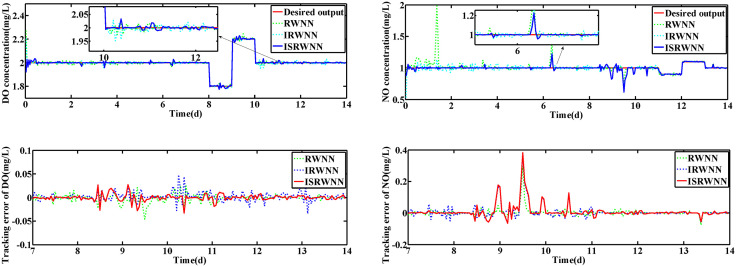
Control results under rainy condition during set-point changed. **(a)** Control results of DO and NO concentration. **(a)** Control errors of DO and NO concentration.

**Fig 15:**
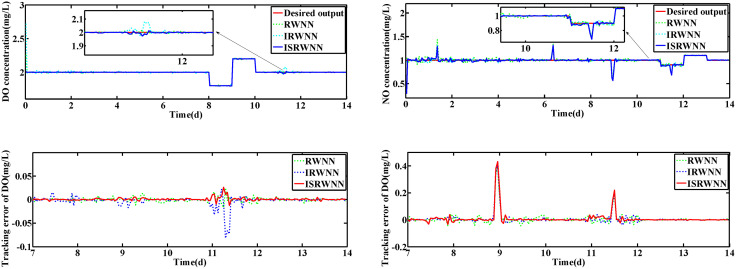
Control results under rainstorm condition during set-point changed.

The operation variables $K_{La5}$ and $Q_{a}$ are shown in Figs 12 and 13, respectively. Obviously, the DO and NO concentration are driven by adjusting the $K_{La5}$ and $Q_{a}$ to track the set-points. The control results are exhibited in Figs 14 and 15. According to the control results, the proposed ISRWNN can obtain higher control accuracy compared with other methods, and the proposed controller can quickly track the changing set value even when there is a step change in the set-points. In the enlarged part, it can be clearly seen that the ISRWNN has a superior control effect and a lower control error.

When the set-points of DO and NO are changed, the performance of the proposed ISRWNN controller is compared with other methods as shown in Table 3. We can observe that the control error of IRWNN is smaller compared with RWNN, indicating that it can solve the coupling problem in WWTP. Meanwhile, the ISRWNNs have the smallest IAE, ISE and ${\text{Dev}}^{max}$ among the compared methods, which illustrate the proposed ISRWNN has better control capability

**Table 3:** Comparison of different control methods for changed set-point.

Condition	Controller	DO				NO			
		*No*.	IAE	ISE	Dev^max^	*No*.	IAE	ISE	Dev^max^
Rainy	ISRWNN	6	0.0029	8.29 × 10^−5^	0.0939	5	0.0093	1.26 × 10^−3^	0.2826
	IRWNN	3	0.0051	3.56 × 10^−4^	0.1031	3	0.0112	6.71 × 10^−3^	0.3226
	RWNN	3	0.0076	6.31 × 10^−4^	0.1664	3	0.0176	2.84 × 10^−3^	0.4245
	RFNN	6	0.0084	5.64 × 10^−4^	0.1778	6	0.3585	4.69 × 10^−2^	1.6809
	RBFNN	5	0.0751	1.71 × 10^−2^	0.1714	5	0.0347	1.84 × 10^−2^	0.9457
Rainstorm	ISRWNN	4	0.0018	4.81 × 10^−5^	0.0469	3	0.0068	1.42 × 10^−3^	0.3313
	IRWNN	3	0.0022	6.59 × 10^−5^	0.0821	3	0.0083	1.76 × 10^−3^	0.3679
	RWNN	3	0.0069	2.67 × 10^−4^	0.0857	3	0.0121	2.65 × 10^−3^	0.4479
	RFNN	6	0.0081	5.66 × 10^−4^	0.0921	6	0.4189	4.24 × 10^−2^	1.2759
	RBFNN	5	0.0141	8.31 × 10^−3^	0.1204	5	0.4516	8.32 × 10^−2^	1.9388

## 5. Conclusions

In this paper, considering the coupling and dynamicity of WWTP, the ISRWNN controllers are designed to simultaneously improve the control accuracy of DO and NO concentration and maintain the stable operation of WWTP. First, to deal with the coupling problem, the input of DO and NO errors are considered comprehensively. Then, the growing and pruning algorithm of wavelet neurons is proposed to solve the dynamicity of WWTP. No matter the set-points is fixed or changed, the ISRWNN can automatically adjust the structure of controller. Experimental results also demonstrate the superiority of ISRWNNs control method compared to other multi-NN controllers.

## 6. Future work

Future work will focus on evaluating the resilience of proposed control method under realistic operating conditions. We will systematically assess the impact of critical disturbances—including measurement noise, microbial activity changes, and inflow fluctuations—on system stability. This analysis will inform the development of enhanced control strategies, potentially incorporating adaptive or robust control elements, to ensure reliable performance in practical applications.
